# Heating up the immune battle: Magnetic hyperthermia against cancer

**DOI:** 10.1016/j.fmre.2024.08.006

**Published:** 2024-09-03

**Authors:** Wangbo Jiao, Lijun Dai, Bin Yan, Yi Lyu, Haiming Fan, Xiaoli Liu

**Affiliations:** a National Local Joint Engineering Research Center for Precision Surgery & Regenerative Medicine, Shaanxi Province Center for Regenerative Medicine and Surgery Engineering Research, Shaanxi Provincial Key Laboratory of Magnetic Medicine, First Affiliated Hospital of Xi'an Jiaotong University, Xi'an 710061, China; b Institute of Regenerative and Reconstructive Medicine, Med-X Institute, First Affiliated Hospital of Xi'an Jiaotong University, Xi'an 710049, China; c Key Laboratory of Synthetic and Natural Functional Molecule of the Ministry of Education, College of Chemistry and Materials Science, Northwest University, Xi'an 710069, China; d Laboratory of Resource Biology and Biotechnology in Western China, Ministry of Education, Provincial Key Laboratory of Biotechnology of Shaanxi Province, Northwest University, Xi'an 710069, China

**Keywords:** Magnetic hyperthermia, Magnetic iron oxide nanomaterials, Nano-scale heat, Reactive oxygen species, Anti-tumor immunity, Hepatocellular carcinoma

## Abstract

Magnetic hyperthermia (MH) utilizes magnetic iron oxide nanomaterials (MIONs) to generate nano-scale heat and boost reactive oxygen species production within cells exposed to an external alternating magnetic field. Unlike conventional thermal ablation therapies that produce heat on a macro-scale, MIONs act as point source of heat inside cells, which enables MIONs-mediated MH to modulate cellular functions and fate with precision in real-time. With key benefits such as deep tissue penetration and the ability to regulate processes in a temporal-spatial and quantifiable manner, MH is now emerging as a new cancer therapy. Most intriguing is the apparent ability for MH to alter specific biological pathways associated with an anti-tumor immune response. Research efforts are now accelerating to render MH applicable to the clinical setting, with the objective of supporting the treatment of common cancers such as hepatocellular carcinoma (HCC). In this perspective paper, we highlight the recent progress made in MH, with a particular focus on its ability to manipulate anti-tumor immune mechanisms and the therapeutic advantages demonstrated thus far for HCC. We explore the current challenges in this field, and provide our perspective on the outlook for MH and its role in cancer treatment.

## 1 Introduction

Cancer ranks as one of the top reasons for death worldwide, seriously impacting global health [1]. Thermal ablation (in which tumor cells are destroyed by the high temperature > 60 °C) is recommended in clinical guidelines for treating various tumor types, such as liver, lung, and kidney cancer [2]. Its minimal invasiveness, remote controllability, and the potential for repeatable treatments contribute to its endorsement as a preferred method in cancer therapy. Unfortunately, existing clinical modalities for thermal-based tumor treatment, such as microwave or radiofrequency thermal ablation, produce heat on a macro-scale and mainly work at the tissue level, resulting in a poor tumor conformity and limited treatment scope. Additionally, there is a high recurrence rate post-ablation. For example, in the case of hepatocellular carcinoma (HCC), the recurrence rate post-ablation can reach up to 70% [3]. Furthermore, over 60% of HCC cases are diagnosed at advanced stages, where the efficacy of thermal ablation markedly decreases due to the challenge in controlling metastasis [3]. There is thus an urgent need for the development of more precise thermal therapies that can specifically target and destroy tumor cells at both early and advanced stages.

**Fig. 1:**
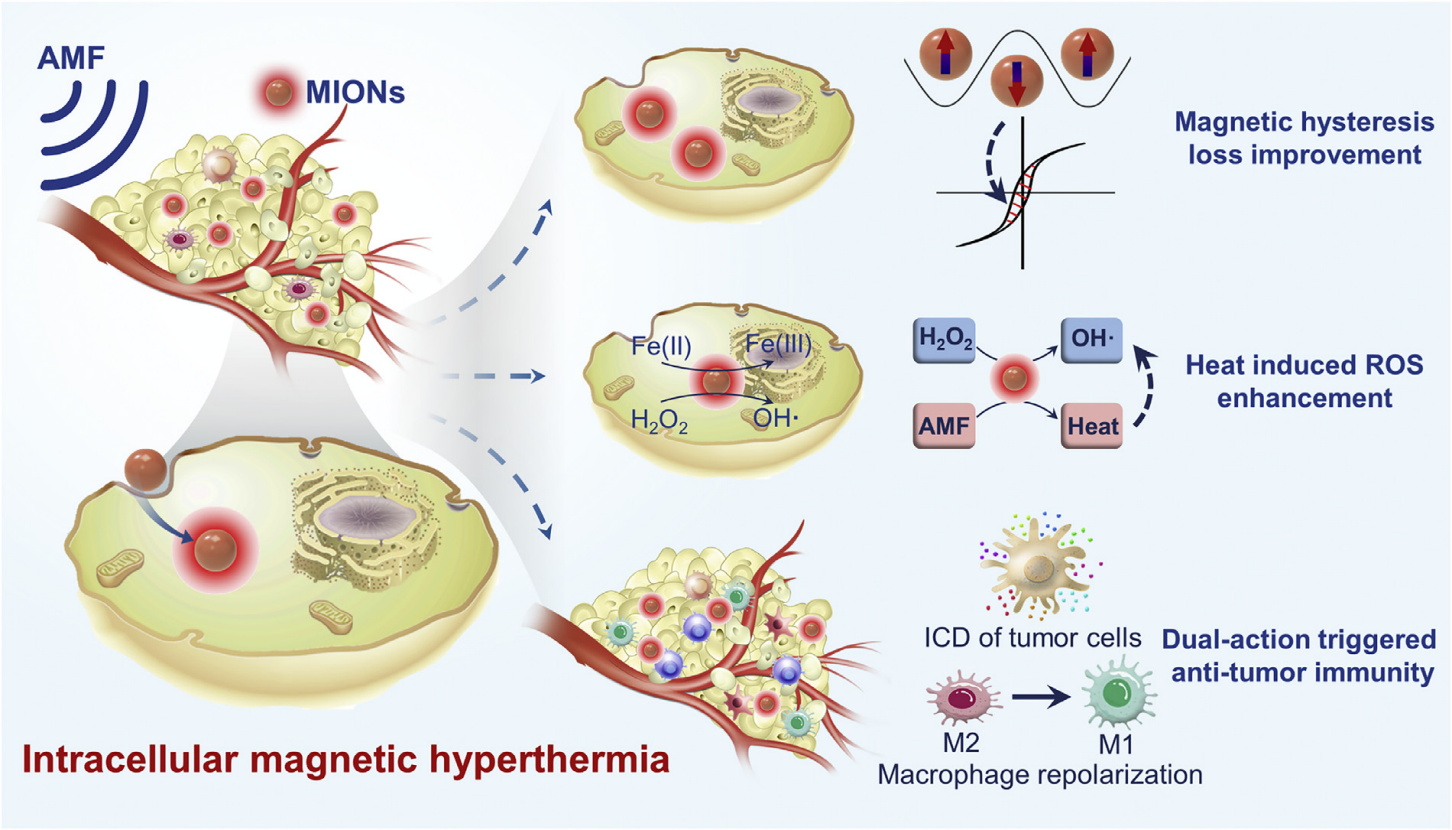
**Key features of intracellular magnetic hyperthermia (MH).** Magnetic iron oxide nanomaterials (MIONs) are efficiently internalized by tumor cells. When exposed to an alternating magnetic field (AMF), MIONs generate nano-scale heat within cells to perform MH. The efficacy of MH is enhanced by improvements in magnetic hysteresis loss and the augmentation of reactive oxygen species (ROS) through heat induction. The intracellular responses initiated by MH, including heat and oxidative stress, have noteworthy implications for anti-tumor immunity. MH has the potential to trigger immunogenic cell death (ICD) in tumor cells and induce the repolarization of macrophages. These immunomodulatory effects contribute to a comprehensive anti-tumor immune response.

Magnetic hyperthermia (MH), an innovative clinical thermal therapy, was approved for clinical use in Europe in 2011 [4]. Magnetic iron oxide nanomaterials (MIONs) are harnessed to generate nano-scale heat and boost the production of harmful reactive oxygen species (ROS) within cancer cells when exposed to an alternating magnetic field (AMF) (Fig. 1), thereby inducing a specific damage to tumor cells while sparing healthy tissue. Unlike conventional thermal therapies that generate heat at the macro-scale affecting broader tissue areas, the point source of heat provided in the present therapy allows for a real-time controlled intervention at the sub-cellular level and modulating cellular functions and fate *in vivo*. Other key features are: it closely matches the tumor's shape, can reach deep into the body, has fewer side effects, and can modulate molecular signaling pathways that activate anti-tumor immune responses (Fig. 1). With appropriate surface modifications, MIONs have an LD50 in animals as high as 2000–6000 mg kg^-1^ [5]. The only reported clinical death associated with MIONs resulted from undiluted intravenous administration [6]. MH is usually administered locally and is handled with extra caution due to the potential for overheating. To date, there have been no reports of medical malpractice relative to MH. MH is especially adept at inducing intracellular heat and oxidative stress responses, along with its influence on various molecular expressions and signaling pathways [[7], [8], [9]], particularly those linked to the immune response (Fig. 1), highlighting its therapeutic potential. This paper explores the distinct characteristics and benefits of intracellular MH, emphasizing its dual role in directly destroying tumor cells and modulating the tumor immune microenvironment. Additionally, we discuss the emerging challenges and suggest potential future directions for the development of MH.

## 2 Intracellular MH induces direct destruction of tumor cells

MH leverages the near-field heat transfer theory to concentrate heat at the nanoscale around the internalized MIONs within tumor cells [10]. This process creates localized high-temperature zones within the tumor cells, while sparing normal tissues and avoiding heat dispersion *via* blood flow. Such focused intracellular heat of MH facilitates the effective modulation of cellular functions and fates. Moreover, MIONs inherently possess peroxidase-like activity, enabling them to catalyze hydrogen peroxide in the tumor microenvironment into ROS, inducing cytotoxic effects in tumor cells [8]. The nanoscale heat generated by MIONs further enhance this peroxidase-like activity [11], leading to a significant increase in intracellular ROS levels, triggering oxidative stress responses and initiating a series of molecular events culminating in the destruction of tumor cells. Thus, by acting as point source of heat inside cells, MION-mediated MH extends the therapeutic effect beyond the tissue level, reaching down to individual cells.

Despite these benefits, clinically applying MH faces numerous hurdles. One of the most critical issues in this field has been the extremely low magnetothermal conversion efficiency, *i.e.*, the specific absorption rate (SAR), approximately 106 W/g [12], of the clinically used superparamagnetic MIONs (Resovist®). Such inefficiency hampers optimal heat generation and precise control of cellular functions and fate, rendering the anti-cancer therapeutic effects less efficient than needed for clinical benefit. The insufficient conversion efficiency also limits the range of magnetothermal effects to macroscopic tissue levels, hindering precise thermal stimulation of biological targets at the micro/nano-scale. Moreover, the mechanisms of action are difficult to trace, making it challenging to understand the underlying processes. Increasing evidence suggests that macroscopic thermal effects cannot fully explain the phenomena observed during MH treatment of tumors, nor can they guide the optimization and advancement of treatment modalities.

Addressing the inefficiencies of current MIONs is crucial for advancing MH therapy. Great efforts are being made in optimizing MION size, composition, morphology, and surface modifications to enhance SAR [[13], [14], [15], [16]]. In 2015, we made a notable advancement by discovering that nanoscale iron oxide with a ring-shaped structure exhibited a unique vortex magnetic domain. This structure provided high magnetic hysteresis area and dispersibility [12], leading to about 50 times more efficient conversion of electromagnetic energy into thermal energy (up to 5,000 W/g) than existing clinical MIONs agents (106 W/g). Our unpublished findings indicate that iron atom-doped MIONs exhibit significantly higher SAR, potentially exceeding 10,000 W/g. This enhanced SAR appears to be associated with the interface between iron atom and iron oxide, although the underlying mechanism requires further investigation. Studies to explore this phenomenon are currently underway.

## 3 MH regulates tumor immune microenvironment

Advanced-stage tumor often presents with extensive local infiltration and systemic metastasis, limiting the effectiveness of current treatments. While immunotherapy, including immune checkpoint blockade therapy, has shown promise in various cancers [17], the highly immunosuppressive tumor microenvironment leads to low responsiveness to these therapies, especially for HCC [18,19]. Thus, precisely modulating both tumor and immune cells within the tumor microenvironment remains a critical challenge.

First noted by Yanase et al. in 1998, MH has potential to elicit anti-tumor immune response [20]. While at the time the underlying mechanisms were not fully understood, we now know that MH in resected HCC samples can release damage-associated molecular patterns (DAMPs) such as calreticulin, high-mobility group box 1 (HMGB1), and adenosine triphosphate (ATP), to activate immune responses [9]. Our work in animal models have further validated these findings, demonstrating that MH can effectively induce immunogenic cell death (ICD) in tumor cells [8,21,22]. These findings led to the development of high-activity, whole-cell tumor vaccines, prepared through *in vitro* MH inactivation in mouse models in 2022. These vaccines have demonstrated efficacy in therapy and in preventing recurrence of homologous tumors following *in vivo* vaccination [9]. By contrast, comparable therapeutic effects were not observed with vaccines prepared using extracellular water bath hyperthermia, despite similar macroscopic temperatures being employed. The significant differences observed underscore the efficacy and importance of intracellular MH-induced ICD of tumor cells in activating anti-tumor immunity.

**Fig. 2:**
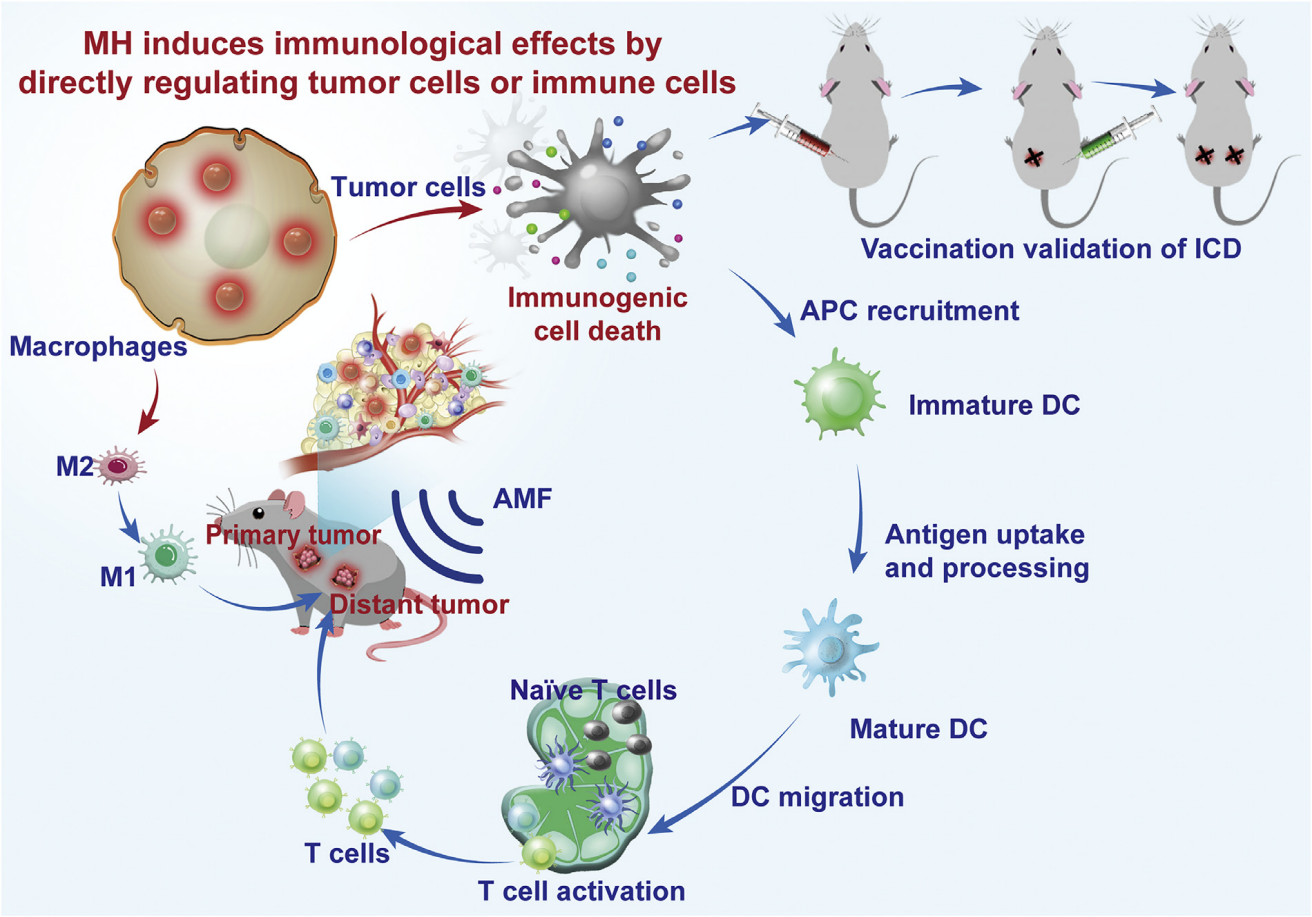
**Immunological effects of intracellular magnetic hyperthermia (MH).** When subjected to an alternating magnetic field (AMF), MH mediated by magnetic iron oxide nanomaterials triggers immunogenic cell death (ICD) in tumor cells and facilitates the recruitment of antigen-presenting cells (APCs), particularly dendritic cells (DCs), to acquire tumor antigens. These matured DCs then migrate to tumor-draining lymph nodes, where they activate T cells. The activated T cells subsequently infiltrate both the primary and distant tumor tissues, initiating an immune response against the tumors. Moreover, MH contributes to the remodeling of the tumor microenvironment by repolarizing tumor-associated macrophages from M2 to M1 phenotype. This polarization shift enhances the anti-tumor immune response. Additionally, the capacity of MH to induce ICD in tumor cells can be harnessed for the preparation of tumor vaccines, serving as an effective approach for adjuvant immunotherapy.

A key aspect of MH is the combined effects of intracellular heating and ROS production [23]. In our mechanistic studies of MH-induced ICD, we found that the amplification of ROS generation induced by MH, is essential for provoking a strong immune response, particularly in the hypoxic tumor microenvironment [8]. This immune response is activated even at temperatures below 40 °C, which are physiologically tolerable and do not cause damage to surrounding healthy tissues [24]. Notably, we found that once MIONs were internalized by tumor associated anti-inflammatory M2-like macrophages, MH could repolarize the cells to a pro-inflammatory M1 phenotype, reshaping the tumor immune microenvironment and promoting T lymphocyte infiltration [8] (Fig. 2). Thus, the synergy between MH-generated heat and increased ROS production contributes to the efficacy of the immune response against cancer cells, making MH an effective strategy for targeting tumors in hypoxic conditions. Furthermore, when combined with PD-L1 blockade, MH exhibits synergistic effects [7]. Notably, in comparison to monotherapy, the combination therapy group showed a significant increase in CD4^+^ and CD8^+^ *T* cell infiltration within the tumors. Concurrently, there was a reduction in the proportion of regulatory T cells and myeloid-derived suppressor cells (MDSCs). This shift in the immune cell landscape led to the complete eradication of *in situ* tumors, with no recurrence or metastasis observed over a 32-day period [7]. This outcome could be particularly promising for HCC, which typically exhibits a low response rate to immune checkpoint blockade therapy [25].

## 4 Future challenges and perspectives

While MH shows significant promise in cancer therapy, its advancement as a novel tumor treatment modality faces several key challenges that necessitate further exploration and development:

(a) **Optimizing intracellular targets**. The intracellular targets of MIONs-mediated MH are crucial but not yet fully defined. MIONs are theorized to be internalized into cells where they localize within the endo-lysosomal system, accumulate in secondary lysosomes [26], and then generate heat in response to AMF and initiate tumor cell death. However, the detailed molecular mechanisms facilitating tumor cell death *via* this pathway are not entirely clear. Specifically, research is focused on understanding how ROS is released from lysosomes and identifying the molecules within the lysosome that play a role in the biological effects of MH. This includes investigating the mechanisms of lysosomal membrane permeabilization and the subsequent release of lysosomal contents, which can trigger various cellular responses, including cell death and immune activation [27]. To harness the full therapeutic potential of MH, further research is also needed to elucidate the specific molecular interactions and pathways triggered by MH. While it's established that caspases play a role in the process, the specific mechanisms through which they signal and lead to cell death, particularly ICD, require further clarification. Furthermore, understanding the differential effects of MIONs-mediated MH across various cellular organelles, including lysosomes, the endoplasmic reticulum, and mitochondria, etc., is crucial. A concerted effort is required to understand and control MIONs’ intracellular positioning. This entails a strategic design approach tailored to these MIONs, enabling the development of MIONs targeted at specific subcellular structures. By doing so, we can significantly advance the precision and efficacy of MIONs-mediated MH in research and therapeutic contexts. In addition, although differences between the effects of intracellular and extracellular hyperthermia have been demonstrated [9], there is still a lack of tools to directly detect the intracellular heat generated by MIONs. We are exploring two approaches to address this. One approach involves delivering a temperature-sensitive fluorescent probe to the same intracellular location as the MIONs and detecting the intracellular heat through the fluorescence changes of the probe. The other, more challenging approach, involves calculating the in-situ temperature of MIONs by inverting the magnetization relaxation harmonic signals released by the MIONs.
(b) **Determining the temporal-spatial MION dynamics within the tumor microenvironment.** A critical aspect of advancing MH in cancer treatment is understanding the distribution dynamics of MIONs in different cell types and regions of tumors after administration. In this regard, it is crucial to note that not only tumor cells, but also various immune and non-immune cells, including tumor-associated macrophages, can internalize MIONs. Tailoring MIONs to either evade phagocytosis by macrophages, targeting other cells, or to enhance uptake by macrophages, can be crucial in effectively reprogramming the tumor immune microenvironment. Such strategic tailoring of MIONs could provide new approaches for manipulating immune responses within tumors, potentially leading to more effective cancer treatments by either directly targeting tumor cells or modulating the activity of macrophages to shift their role from tumor-supporting to tumor-fighting. More interestingly, in our research, we've found that intracellular MH can simultaneously reduce the levels of CD47 in tumor cells and SIRPα in macrophages, enhancing antitumor immunity [22]. By modulating the CD47-SIRPα pathway, which is a crucial checkpoint for macrophage phagocytosis, MH presents a multifaceted and effective strategy in the fight against cancer. This dual down-regulation disrupts the "don't eat me" signal that tumor cells often exploit to evade immune destruction, thereby facilitating macrophage-mediated clearance of cancer cells. Moreover, the interaction and impact of MH on other key immune cells, such as granulocytes, B cells, and natural killer (NK) cells, is another area in need of further exploration. Investigating these dynamics will not only broaden our understanding of how MIONs-mediated MH influence the complex interplay within the tumor microenvironment but also guide the development of more precise and efficacious MH strategies for cancer therapy.
(c) **Synergizing MH with chemotherapy.** As chemotherapy remains a cornerstone in treating advanced-stage cancer, integrating MH with chemotherapy seems to be a logical combined therapeutic approach. Current challenges with chemotherapy encompass unacceptable side effects and the development of drug resistance. MIONs can be engineered to deliver chemotherapeutic agents directly to tumor cells. This targeted approach ensures efficient drug delivery to the intended site, minimizing off-target effects and potentially reducing the side effects associated with conventional chemotherapy [28]. Early studies also indicate that MH may reduce the expression of drug-resistance proteins in tumor cells [29]. This finding suggests a potential dual benefits of MH as an adjuvant to chemotherapy: not only does MH directly attack tumor cells, but it may also sensitize them to chemotherapy by reversing drug resistance. This approach could significantly bolster treatment efficacy, offering a new horizon in the battle against cancers especially advanced-stage cancer.
(d) **Developing autologous tumor vaccines by MH.** The use of MH in inactivating tumor cells offers an innovative pathway for developing autologous tumor vaccines. Such vaccines, created from a patient's own tumor cells, represent a highly personalized form of immunotherapy [30]. This approach holds promise for enhancing the effectiveness of immunotherapy, particularly in tumors that show a limited response to checkpoint blockade therapies. The process of MH, mediated by MIONs, is adept at inducing ICD, thereby potentially improving antigen presentation in these vaccines. A key advantage of this method is the ability to swiftly separate MIONs from the tumor vaccine formulation through magnetic separation [31,32]. This approach ensures minimal exposure to exogenous substances, thereby maintaining the integrity and safety of the vaccine. This approach could mark a significant step forward in personalized cancer treatment, offering a tailored therapy that harnesses the patient's own tumor cells to stimulate a more effective immune response against tumor.
(e) **Advancing the next-generation MH system.** The current landscape of MH, encompassing both the MIONs-based agents and the associated equipment, requires significant development enhance thermal efficacy and minimize potential off-target effects. Another vital area for improvement in MH treatment is the focusing of the AMF. Currently, AMF is applied in an unrestricted manner, leading to a rapid divergence of the magnetic field. Narrowing the spread of AMF through the use of a static magnetic field is essential for achieving targeted therapy, thereby ensuring that the therapeutic effects are concentrated on the tumor site while minimizing potential harm to the surrounding healthy tissues. The development of a new generation of high-performance agents for MH, along with equipment that generates restricted, focused magnetic fields, is crucial for boosting the efficacy and safety of this therapeutic modality. This upgradation of MH agents and equipment will not only increase the therapeutic potential of MH but also broaden its applicability in treating various forms of cancer, including HCC.

## 5 Concluding remarks

Over the last 10 years, there has been a significant enhancement in the comprehension of MH. Although the concept was introduced earlier, initial studies did not emphasize its intracellular aspects as much. The nanomedicine field, recognizing the unique potential of MH, has since conducted extensive research. This article has delved into the molecular dynamics occurring within tumor cells at an intracellular level and discussed the immunological effects MH triggers against tumors, uncovering valuable insights into its therapeutic potential. Concurrently, the clinical application of MH has expanded beyond its initial approval in Europe for treating glioblastoma, now reaching into trials for prostate cancer. As of February 2024, there is an active clinical trial in Poland exploring the integration of MH with radiotherapy and chemotherapy specifically for glioblastoma multiforme treatment (NCT06271421). This trial is currently in the phase of recruiting participants, indicating the growing interest and potential for MH in diverse therapeutic contexts. With continuous progress in understanding the mechanisms behind MH and advancements in technology, the upcoming decade holds the potential for significant expansion of MH into more clinical trials. This could lead to its approval for treating HCC and other types of tumors. There's a sense of optimism in the medical community that MH will yield positive results, especially when combined with other treatments like radiotherapy, chemotherapy, and notably, immunotherapy. This anticipation sets the stage for MH to play a pivotal role in the next generation of tumor thermotherapy, marking an innovative approach in cancer treatment.

## Declaration of competing interest

The authors declare that they have no conflicts of interest in this work.
